# Multiplexed chemostat system for quantification of biodiversity and ecosystem functioning in anaerobic digestion

**DOI:** 10.1371/journal.pone.0193748

**Published:** 2018-03-08

**Authors:** Diane Plouchart, Kim Milferstedt, Guillaume Guizard, Eric Latrille, Jérôme Hamelin

**Affiliations:** LBE, Univ Montpellier, INRA, Narbonne, France; Purdue University, UNITED STATES

## Abstract

Continuous cultures in chemostats have proven their value in microbiology, microbial ecology, systems biology and bioprocess engineering, among others. In these systems, microbial growth and ecosystem performance can be quantified under stable and defined environmental conditions. This is essential when linking microbial diversity to ecosystem function. Here, a new system to test this link in anaerobic, methanogenic microbial communities is introduced. Rigorously replicated experiments or a suitable experimental design typically require operating several chemostats in parallel. However, this is labor intensive, especially when measuring biogas production. Commercial solutions for multiplying reactors performing continuous anaerobic digestion exist but are expensive and use comparably large reactor volumes, requiring the preparation of substantial amounts of media. Here, a flexible system of Lab-scale Automated and Multiplexed Anaerobic Chemostat system (LAMACs) with a working volume of 200 mL is introduced. Sterile feeding, biomass wasting and pressure monitoring are automated. One module containing six reactors fits the typical dimensions of a lab bench. Thanks to automation, time required for reactor operation and maintenance are reduced compared to traditional lab-scale systems. Several modules can be used together, and so far the parallel operation of 30 reactors was demonstrated. The chemostats are autoclavable. Parameters like reactor volume, flow rates and operating temperature can be freely set. The robustness of the system was tested in a two-month long experiment in which three inocula in four replicates, i.e., twelve continuous digesters were monitored. Statistically significant differences in the biogas production between inocula were observed. In anaerobic digestion, biogas production and consequently pressure development in a closed environment is a proxy for ecosystem performance. The precision of the pressure measurement is thus crucial. The measured maximum and minimum rates of gas production could be determined at the same precision. The LAMACs is a tool that enables us to put in practice the often-demanded need for replication and rigorous testing in microbial ecology as well as bioprocess engineering.

## Introduction

Lack of replication [1] or application of a suitable experimental design [2] is a recurring problem in experimental work in process engineering and microbial ecology. It is often caused by technical difficulties or the availability of material. The easiest way of replicating experiments in the laboratory from a technical point of view is by multiplying batch experiments [3]. An important characteristic of a batch experiment is that nutrients are fed to the system once as an initial pulse. This pulse is consequently degraded by the microbial community that is faced with changing environmental conditions with less and less available nutrients and the potential accumulation of metabolites over time. Batch experiments are widely used and perfectly suited, when for example testing methane and hydrogen production as a function of substrate pretreatment [4], assessing the effect of experimental protocols [5], characterizing key species involved in specific activity [6,7], or studying transient phenomena like the degradation of crude oil spills [8]. In these situations, the batch set-up mimics well the environmental process in question. A continuously operated reactor system like a chemostat is better suited to reproduce the constant exposure of a microbial community to permanently replenished contaminants or nutrients, as for example in soil around a leaking oil tank, in a wastewater treatment plant or an anaerobic digester. Furthermore, the controllable settings in chemostats such as substrate concentration, hydraulic retention time or temperature may make it possible to link molecular data from ‘omics’ technologies to environment parameters [9] and allow studying the contributions of microbial diversity, community dynamics, and microbial interactions to process stability [10].

Replication or a more complex experimental design can easily require the operation of several reactors in parallel over extended periods. However, operating numerous chemostats over long times is technically challenging. It requires technical expertise and a significant amount of manpower, going along with increasing costs and complexity of the set-up. These factors make experiments in parallel for example in anaerobic digestion difficult where reactor operation may last several months. Several commercial solutions exist for multiplexing chemostat operation. However, the prices of these systems is typically cost-prohibitive for publically funded academic research labs or are missing an important property as for example the suitability to be operated as anaerobic digesters with continuous quantification of ecosystem performance.

The objective was to develop an affordable and versatile system, allowing the operation of a maximum number of chemostats in parallel by a single person. With the Lab-scale Automated and Multiplexed Anaerobic Chemostat system (LAMACs) introduced here, the automated measurement of biological activity in biogas-producing ecosystems is feasible. In this study, the design, application range and limits of the LAMACs are presented, as well as a first application on anaerobic digestion and its suitability for generating biomass samples for molecular ecological purposes.

## Materials and methods

### Design of the LAMACs

**Fig 1:**
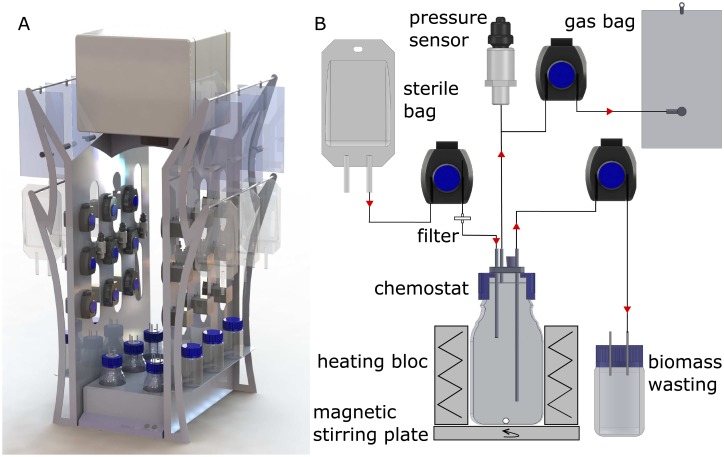
General view of the LAMACs and of components of one chemostat in the LAMACs module. (A) 3D view of a LAMACs module, i.e., six chemostats. The electric box containing controlling cards is on the top of the module and above any source of liquids to prevent electric failure in case of leakage. A waterproof stirring plate is underneath the heating block containing the six reactor vessels. Three peristaltic pumps are aligned above each vessel. The upper range of bags is for biogas collection; the lower range serves as substrate reservoirs. The dimensions of one module are 50 cm width × 52 cm length × 100 cm height. (B) Schematic view of a chemostat detailing the use of the three-hose connector. One port is used as inlet for feeding, and two ports as outlet for biomass wasting and degassing. One port for manual sampling is sealed with a rubber stopper. A 0.45 μm pore-size filter is placed in the feedline ahead of the reactor to prevent contamination of the medium.

One module of the LAMACs is composed of six chemostats as shown in Fig 1A of which each reactor can be operated independently, thus allowing flexibility in the experimental design. Any number of these modules can be operated in parallel, limited only by the available manpower. The dimensions of one module are 50 cm width × 52 cm length × 100 cm height. It fits well on a standard laboratory bench. Temperature is controlled using a custom-made aluminum heating block (Garaud, Carcassone, France) to snugly house 250 mL borosilicate graduated laboratory bottles with standard GL45 threading that serve as reactor vessels. The thermostat integrated in the aluminum block allows setting a temperature range from room temperature to 60 °C. Using Lab Guard II temperature sensors (AES, Chemunex, France), homogeneity of the heat distribution was verified. A standard waterproof magnetic stirring plate was placed underneath the heating block (Variomag Multipoint 6, Thermo Scientific, Waltham, United-States). Three peristaltic pumps with stepping motors (FZ10, A2V, Gazeran, France) were connected to each chemostat for automated substrate loading, biomass wasting and degassing (Fig 1B), thus 18 pumps per module. All peristaltic pumps were calibrated before use (S1 Fig). Peristaltic pumps were connected to a controller module (TMCM 6110, Trinamic, Hamburg, Germany) and were piloted via a free software (TMCL-IDE, Trinamic, Hamburg, Germany).

The feed, waste and degassing tubes were connected to the 250 mL reactor vessels through stainless steel three-hose connectors inserted into GL45 caps with aperture. The custom-made cap connector (Garaud, Carcassone, France) is gas tight through a rubber joint between the reactor and the insert under the recommended pressure range of the reactor vessels (maximum of 1.5 bar). The ends of the three-hose connectors are thickened to increase tightness of the tubes. The inner diameter of the connector tubes is 2 mm. In addition to the three-hose connector tubes, there is one additional 5 mm hole in the cap connector. This hole is sealed with a 10 mm long conical rubber stopper and serves as septum for direct sampling, e.g., of reactor content for metabolite analyses. All parts in contact with the reactor interior can be autoclaved for sterile operating conditions.

Sterile bags of 100 mL (Easyflex+, Macopharma, Mouvaux, France) were used as nutrient reservoirs. These bags can be easily filled, stored and replaced. Media bags were filled aseptically with enough feed for one week of operation and connected to the reactor. Effluent from the reactor was temporarily stored in 100 mL graduated laboratory bottles with GL45 threading and daily removed for analyses. Biogas was collected in 100 mL Tedlar^®^Gas Sampling Bags (Sigma-Aldrich, St. Louis, USA). Tygon pump tubing (2 × 4 mm R3603, Saint-Gobain, Courbevoie, France) was used for feeding and wasting. Norprene^®^ tubing (6404 LS14, Saint-Gobain, Courbevoie, France) was used for gas management. Pump tubing was replaced on a weekly basis.

Using pressure sensors, the system is able to detect biological activity directly by measuring the production of biogas in a closed environment. One pressure sensor per chemostat was used with a sensitivity of 0.0015 bar and an accepted maximum pressure of 3.4 bar (PX2EN1XX050PAAAX, Honeywell, New Jersey, United-States). It was placed in line between the chemostat and the peristaltic pump used for degassing. All pressure sensors were calibrated before use (S2 Fig). The pressure sensor tolerates operation in humid environments. Pressure data were constantly monitored by the same controller and software as for the peristaltic pumps. The interval for pressure data collection was set to 20 s, i.e., every 20 s, the current pressure in the reactors was recorded using a custom code written in Python 2.7 (https://www.python.org/). For the experiments described here, degassing began when a pressure of 1.2 bar was reached. Gas was pumped out of the system until the pressure fell to 1.05 bar.

All the equipment for the construction of one module (six reactors) cost about 7000€ in 2017 and are detailed in the Table 1 below and in S1 Table where supplier references are added. About half of the budget was used for pumps and sensors with their power supplies. The remainder was used for the chassis, magnetic agitator, heating bloc and three-hose tube connector. Operational costs were estimated to be around 400€ for a one month experiment of six reactors in an anaerobic digestion process, mostly used for consumables, e.g., gas bags (reusable), tubing, sterile bags for substrate and sterile filters. Some of the equipment described in Table 1 as for example the dry bed heating bloc or the magnetic stirring plates can be replaced by less expensive alternatives.

**Table 1:** Detailed prices of major components required for the construction of one LAMACs module containing six reactors. Detailed references for these items can be found in S1 Table.

	Equipment for one module LAMACs -6reactors	cost for 6 reactors (€)
**bottling**	6 Stainless steel three-hose connectors	222
	250 mL pyrex bottles, cap connectors, magnetic stirrer	39
	subtotal	**261**
**temperature regulation**	Heating bloc	807
	Temperature regulator	140
	subtotal	**1169**
**feeding and biomass wasting**	12 peristaltic pump with stepping motors (FZ10)	1320
	Controller module (TMCM 6110)	615
	Motors power supply	80
	subtotal	**2015**
**pressure measurement**	6 peristaltic pump with stepping motors (FZ10)	660
	6 Pressure sensor (PX2EN1XX050PAAAX, Honeywell)	380
	Pressure sensor power supply	50
	subtotal	**1090**
**unit assembly**	Chassis	707
	Electric jacket	30
	Laptop computer	600
	subtotal	**1337**
**mixing**	Magnetic stirring plate (Variomag Multipoint 6)	**1060**
	**Total**	**6932**

### Data analyses

All pressure data and statistics were analyzed in the R software environment, version 3.3.1 [11]. Raw pressure data were acquired as absolute pressure readings in the range of 1.05 to 1.2 bar. Pressure data were converted into biogas volume in three different signal processing steps: first, pressure drops due to automatic degassing after biogas accumulation (1.2 to 1.05 bars) were removed to obtain a curve of cumulated absolute pressure. Secondly, occasional sudden sharp increases or decreases in pressure caused by technical problems (i.e., gas leakage, liquid sampling) were removed so that they did not contribute to the accumulated signal. The limits for this removal were pressure spikes of +/- 10 mbar∙min^-1^ for one measurement, e.g., a 20 s time interval. The corrected data were then converted to normalized biogas volume under standard conditions, i.e., 293.15 K and 1.013 bar. Biogas production rates were then estimated from linear regressions.

The precision of pressure sensors was evaluated in a dedicated experiment with four reactors under anaerobic digestion conditions over several days.

### Long-term operation of the LAMACs

Digestate from three different origins were sampled from pilot-scale, solid-state mesophilic anaerobic digesters that had been operated under identical environmental conditions for 1.5 years [12]. One anaerobic digester received readily biodegradable substrates (grass and carrots), the second digester received intermediately biodegradable substrate (grass and manure) and the third digester received slowly biodegradable substrates (manure and dung). Digestates from these reactors were anaerobically conditioned for one week prior the experiment to allow the degradation of the remaining organic matter. They were used to inoculate four replicated anaerobic reactors per inoculum at a volatile solids (VS) concentration of 5 gVS∙L^-1^. The three inocula are named in this manuscript ‘INOC A’, ‘INOC B’ and ‘INOC C’, respectively. The reactors were operated in continuous mode, at a constant hydraulic retention time of 15 days over nine weeks under mesophilic conditions (37 °C). The reactors were fed with a complex substrate made of polymers and diverse sugars as carbon sources (S2 Table) with an organic loading rate expressed as chemical oxygen demand (COD) of 1.33 gCOD∙L^-1^∙d^-1^. During weeks 4 to 6, the organic loading rate was halved with a simple substrate made of monomers (S2 Table). Biogas production was recorded online and expressed as a weekly production rate in mL∙d^-1^∙gCOD_added_^-1^. Biogas composition was measured on a weekly basis, as well as the pH measured with hand-held pH meter and electrode (SG23 with Inlab Expert Go-10m-ISM, Mettler Toledo InLab, Greifensee, Switzerland).

Volatile fatty acids (VFA) were sampled directly from the chemostat, filtered (0.45 μm) and injected in a gas chromatograph (CPG Clarus 580, Perkin Elmer, USA) equipped with an auto-sampler, with an Elite-FFAP crossbond^®^carbowax^®^ 15 m column connected to a flame ionization detector at 280 °C, using nitrogen as carrier gas at a flow rate of 6 mL∙min^-1^.

A total of 12 mL per day of reactor content was removed from the reactor in increments of 0.5 mL∙h^-1^. This liquid was collected in a designated bottle for offline analyses, e.g., measurements of total suspended solids, or for the extraction of DNA for community composition analyses. Volatile solids were measured according to standard methods of the American Public Health Association [13]. The soluble chemical oxygen demand was measured from 2 mL of reactor content after centrifugation and filtration (0.45 μm) using prefilled COD tubes (Aqualytic 420721 COD Vario Tube Test MR, 0–1500 mg∙L^-1^, Aqualytic, Dortmund, Germany), placed in a HACH COD reactor at 150 °C for 2 h. COD concentrations were determined photometrically at 620 nm (Photometer MultiDirect, Aqualytic, Dortmund, Germany).

250 μL of biogas were manually sampled directly from headspace of the reactors. The biogas composition was measured with a gas chromatograph (Clarus580, Perkin Elmer, Waltham, USA) equipped with a thermal conductivity detector and two columns: RtQBond to split H_2_, O_2_, N_2_, CH_4_ and RtMolsieve (5Å) to separate CO_2_. The carrier gas was argon at an initial pressure of 3.5 bar. The temperature was 60 °C in the oven, 250 °C in the injector and 150 °C for the detector. The gas chromatograph was calibrated using a standard gas mixtures (399152, Linde, Munich, Germany) containing 25% CO_2_, 2% O_2_, 10% N_2_, 5% H_2_ and 58% CH_4_.

Each week, the different production rates of the replicated inocula were statistically compared with a Kruskal-Wallis test. When the test was significant, a Dunn post-hoc test was performed to account for multiple comparisons of independent samples, using the function ‘posthoc.kruskal.dunn.test‘ from the R package ‘PMCMR’ (version 4.1) [14].

## Results

### Range of operating conditions of the multiplexed chemostats

Each of the six chemostats in a LAMACs module can be operated independently (Fig 1). While the temperature and mixing regime is fixed for the entire module, working volume and flow rates can be individually assigned to each reactor. The range of working volume is from 50 to 200 mL. The upper limit is determined by the height of the heating block. The lower limit is determined by the maximum length of the biomass wasting tube that still allows free movement of the magnetic stir bar.

The dilution rate can vary from a zero wastage mode, i.e., fed-batch mode, to a rate compatible with the maximum doubling time of *Escherichia coli* of 20 minutes. The dilution rate can be adjusted by tuning the working volume and the flow rates of the peristaltic pumps. The LAMACs is operated in quasi-continuous mode because of the periodic nutrient addition. Regular pauses are required to prevent overheating of the peristaltic pumps. In our application, the frequency of pulse additions of 24 times a day is high compared to the hydraulic retention time of the system, especially when working in anaerobic digestion with hydraulic retention times of at least 15 days.

**Fig 2:**
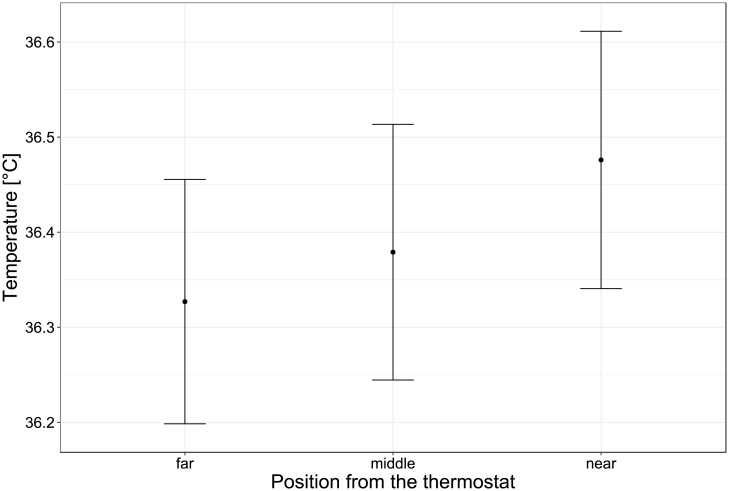
Stable temperature of the LAMACs during operation. Average temperature and standard deviation over three days of operation at three different positions in the heating block. Data points were recorded every 15 minutes, with 280 total data points.

As temperature influences biological activity, the temperature homogeneity between reactors positions was considered a priority and investigated in detail. At three distances from the heating probe, temperatures inside reactors were monitored over three days (Fig 2). The average temperature varied from 36.33 °C to 36.48 °C with a standard deviation of 0.13 °C.

### Sensitivity of performance measurement

**Fig 3:**
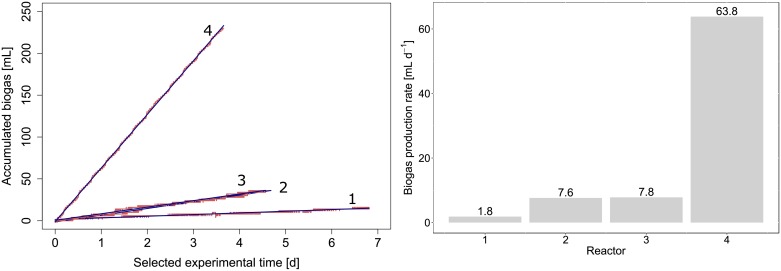
Biogas production of four anaerobic digesters measured by the LAMACs. A) Actual accumulated biogas over time (red curves) and linear regressions (blue lines) B) Biogas production rates derived from linear regressions.

To assess reliability of pressure determination and particularly low production rates, a dedicated experiment was performed with four anaerobic digesters with marked performance differences. In Fig 3A, biogas accumulation over several days of stable reactor operation is shown. Biogas production rates for the same periods are displayed in Fig 3B, ranging from 1.8 mL∙d^-1^ (reactor 1) to 63.8 mL∙d^-1^ (reactor 4), the latter being the highest observed production rate in the experiment. Reactors 2 and 3 had similar but statistically distinguishable production rates of 7.6 and 7.8 mL∙d^-1^, respectively. The lowest detected biogas production rate of 1.8 mL∙d^-1^ (Reactor 1) was highly significantly different from signal at ambient pressure without biogas production (Student test, p-value <0.001). Noise introduced by the pressure sensors was therefore negligible. The linearization of pressure increments after degassing events presented small pressure increases visible in Fig 3, which likely result from gas-liquid transfer of dissolved CO_2_ and CH_4_ after the pressure change through degassing. This is not considered a problem for the data analysis. Linear regressions of biogas volume over time allowed the precise determination of biogas production rates (Table 2), with coefficients of determination (r^2^) above 0.945 and standard error of the slope below 0.01 mL∙d^-1^.

**Table 2:** Linear regression of biogas accumulation over time.

Reactor number	Slope [mL∙d^-1^]	r^2^	Standard error [mL∙d^-1^]	number of data points
Reactor 1	1.8	0.930	0.003	24486
Reactor 2	7.6	0.983	0.007	22641
Reactor 3	7.8	0.983	0.008	19182
Reactor 4	63.8	0.999	0.009	17862

### Long-term operation of the multiplexed chemostats

The LAMACs was designed for a long-term operation of numerous anaerobic digesters at a time. The technical objective of this experiment was to challenge the continuous operation of the LAMACs over a long period, i.e., more than two months. The LAMACs was tested by incubating three inocula in four replicates, therefore twelve anaerobic digesters over nine weeks.

**Fig 4:**
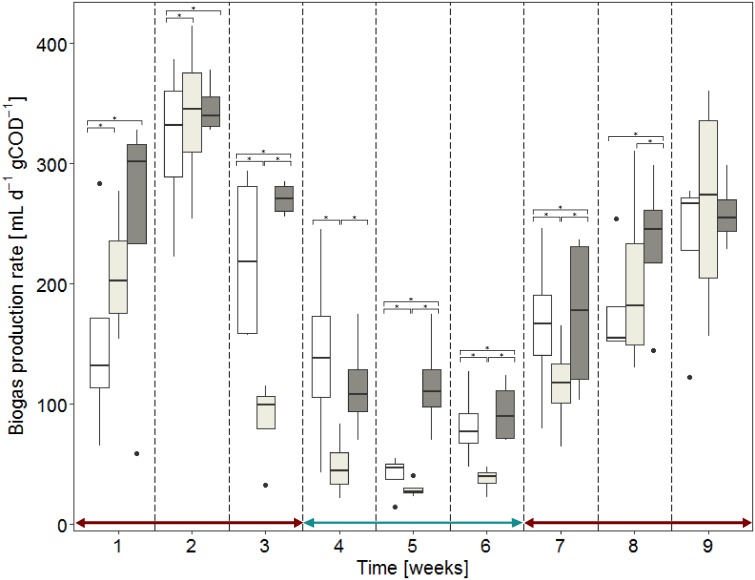
Development of average weekly biogas production rates over time for anaerobic digesters with three different inocula. Four replicated anaerobic digesters were operated per condition. White boxes stand for the INOC A condition, light gray boxes for INOC B and dark-gray boxes for INOC C. The Kruskal Wallis test was significant in the first eight weeks and the Dunn post hoc test for pairwise multiple comparison displays inocula differences with ‘*’ symbols. In weeks 4 to 6, the simple substrate was used instead of the complex substrate.

Biogas production rates were reported in Fig 4 as a function of inocula origins and time. Results are presented on a weekly basis. Despite having more highly resolved data available, this interval was chosen because soluble COD, hydrogen, methane and biomass production required for establishing a COD balance were measured once a week.

Biogas production rates ranged from 14.6 to 414.4 mL∙gCOD^-1^∙d^-1^ (Fig 4 and S3 Table). The biological variability of the four replicated reactors is displayed by the size of the boxes in box plots in Fig 4. This intrinsic biological variability was smaller than the variation of performances over time due to operating conditions, and less than the differences between inocula sources. Performances were normalized by the added substrate to be able to compare periods with different substrate loads. The performance decreased when the substrate load was decreased between week 4 and week 6. For example, the inoculum originally fed with slowly biodegradable substrates (INOC C, dark-gray color in Fig 4) had an average production rate of 282 mL∙gCOD^-1^∙d^-1^ over the first three weeks and then dropped to 108 mL∙gCOD^-1^∙d^-1^ between week 4 and week 6 when the load was reduced. The performance rose again to 222 mL∙gCOD^-1^∙d^-1^ between week 7 and week 9 after increasing again the load. Although similar trends were observed, the three inocula sources performed differently most of the time (Kruskal Wallis test, p-value<0.05).

**Fig 5:**
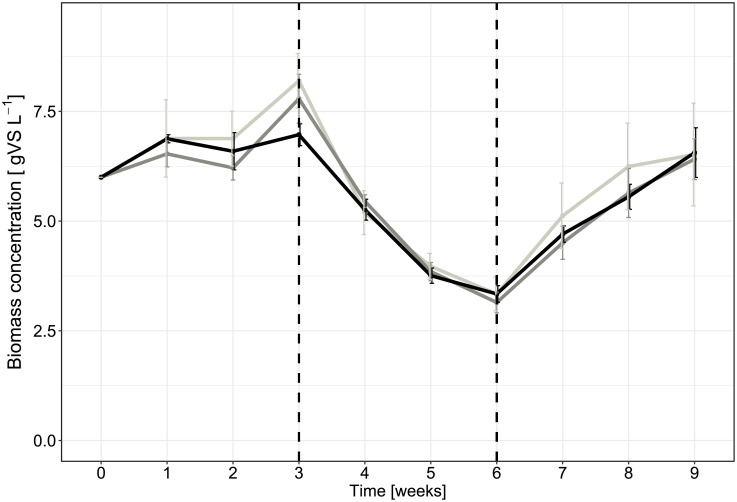
Dynamics of biomass concentration for twelve anaerobic digesters over nine weeks. Biomass concentration is expressed as volatile solids. A color code was applied by inoculum origin; Light gray stands for INOC A replicates, dark-gray stands for INOC B replicates and black stands for INOC C replicates. During weeks 4 to 6, a simple substrate with halved loading rate in terms of COD was applied to the reactors.

While differences in the biogas production rates were shown according to the origin of the inocula (Fig 4), similar biomass concentrations expressed as volatile solids (Fig 5) and total volatile fatty acid concentrations (S3 Fig) were observed in all twelve reactors. Significant differences in biogas production rates (S3 Table) were thus related to the specific activity of microbial communities since total biomass did not differ between the different inocula sources (Fig 5). These changes in biogas production rates may be explained by the shift of substrate concentration and composition every three retention times. These shifts can affect the ability of active microorganisms to degrade the actual substrate.

## Discussion

Operating multiple anaerobic digesters in continuous mode is labor intensive. Braun et al. (2015) worked with 12 manually operated continuous reactors with a working volume of 400 mL and a hydraulic retention time of 20 days over 100 days for testing the fate of polycyclic aromatic hydrocarbons with three different microbial communities [15]. Operation of these reactors required full-time attention. The apparent need for a multiplexed solution, reducing maintenance and operation requirements is obvious, bearing in mind that the required number of reactors to be operated in parallel may exceed 12 to address many scientific challenges. There are highly multiplexed commercial solutions available for example the AMPTS system by Bioprocess Control [16] that enables the operation of 15 batch reactors in parallel with automated real-time methane flow monitoring. This multiplexed solution allows the use of complex experimental designs. For example, Sierocinski et al. (2017) presented an application of the AMPTS by testing the effect of community coalescence on ecosystem performance in a gradient of 1 to 12 combined methanogenic communities [17]. However, AMPTS allows only batch operations where active and dead cells stay in the system. In continuous mode, only actively multiplying cells can maintain their presence when facing washout. Full-scale wastewater treatment plants and also anaerobic digesters are operated in continuous mode. When studying these processes at the laboratory scale from an ecological and bioprocess engineering point of view, mimicking continuous operation is thus essential.

Starting experiments sequentially is one way around the use of a multiplexed continuous reactor system. When using pure cultures, it is possible to repeat experiments or to test alternative experimental conditions even of a complex experimental design one at a time, as the starting point of the experiment is presumably reproducible. When working with complex microbial communities, the assumption of a reproducible starting point is not supported as it is known [18,19] that the microbial community structure and ecosystem performance of an inoculum cannot be easily conserved [5,20,21]. It is therefore necessary to conduct all experiments belonging to an experimental design at the same time. Knowing the limits of batch operation, it may be desirable to conduct a follow-up experiment to the study of Sierocinski et al. (2017) [17], testing the effect of substrate composition on the performance of coalesced microbial communities in continuous operation. Already for a relatively simple design of this experiment, 30 reactors operated in parallel are required when three different substrates and five different inocula with their respective mixtures were considered. This number of continuous reactors is achievable only if most of the operation is automated and if the reactor volume is not too large to minimize the time-consuming preparation of complex mixtures of organic substrates.

Without even addressing the problem of cost, the commercial system from Anaero Technology (www.anaero.co.uk/) appears most closely related to the LAMACs but has a more than four times larger reactor volume requiring the preparation and storage of larger amounts of feed. While this comparably large reactor volume may be advantageous for various applications, in many other situations (e.g., working with sterile feed), large reactor volumes are unmanageable. In contrast, miniaturization and multiplexing of experimental systems has long been done using flow cells with a volume of as little as 5 mL, for example in biofilm studies [22]. More recently, multiplexed chemostats with small volumes on the order of 10 mL [23–25], or even truly microfluidic devices with working volumes of around 2 μL have become available [26]. However, we consider for our purposes the lower limit of acceptable reactor volume to be approximately 50 mL. With this volume, in combination with a sufficiently short hydraulic retention time, enough microbial biomass is generated for off-line measurements of, for example, metabolites, biomass concentration and or for microbial community analyses [27]. With the settings that were used with the LAMACs in this study, 12 mL∙d^-1^ of effluent is available for off-line measurements. Currently, the effluent is stored at ambient temperature with the potential exposure to oxygen. These conditions may possibly induce changes in effluent quality, e.g., COD consumption through heterotrophs, growth of biomass and volatilization of organic acids. Refrigeration of the effluent can be envisioned as future improvements to the LAMACs.

A recurring need from experimentalists [28–30] as well as modelers in microbial ecology [31,32] is the ability to interconnect ecosystems for testing effects on ecosystem performance by migration, washout or connectedness to a larger metacommunity. Connected chemostats in series may also mimic digestive tract topology [33–36]. This required flexibility is built into the design of the LAMACs where each chemostat can be either operated individually or in series by connecting it to other reactors. The only constrain is a fixed temperature between room temperature and 60 °C and a fixed mixing regime for all reactors within a module (Fig 1).

In the current configuration as anaerobic digester, ecosystem performance is immediately accessible through the rate of biogas production. This parameter of crucial importance for biodiversity and ecosystem functioning experiments is measured directly as pressure development in the LAMACs (Fig 1). The reliability of the LAMACs has been demonstrated in data acquired over the period of nine weeks by showing the biological activity in replicated reactors and three types of inocula (Fig 4). Ritter counters (Dr.-Ing. Ritter Apparatebau GmbH & Co. KG, Bochum, Germany), operating by volume displacement, are frequently used for anaerobic digestion, but they require a minimum flow rate of 24 mL∙d^-1^ (https://www.ritter.de/en/products/milligascounters/). Ritter counters were not adapted to the LAMACs because much smaller gas production rates can be expected, and were observed with 1.8 mL∙d^-1^. The experimentally determined observed maximum biogas production rate of 415 mL∙gCOD^-1^∙d^-1^ (Fig 3 and Table 2) should not be considered as the system’s maximum as much more frequent degassing could be accomplished than the two or three degassing events per day that were observed here. Thus, the LAMACs can be used with a variety of substrates ranging from slowly to highly degradable. Equally, it is possible to use the LAMACs with a more active biomass, i.e., for biohydrogen or ethanol fermentations.

A manageable system of continuous lab-scale chemostats was created, tested and validated for microbial ecology and bioprocess engineering applications. In a recent experiment, one person was able to operate five LAMACs modules simultaneously (S4 Fig), i.e., 30 anaerobic digesters, over a twelve-week period. This operation is a significant improvement compared to previous studies [15] and was made possible by automation of degassing, feeding and biomass wasting, as well as miniaturization. Samples collected during the experiment were suitable for molecular analyses as we presented in S5 Fig, with the quantification of *Bacteria* by quantitative PCR. The full experiment will not be detailed here but serves as proof of concept of the LAMACs. One LAMACs module with six reactors can be built for less than 7000 € (Table 1), thus less than 1200 € per reactor. One LAMACs reactor is four times cheaper than the most comparable commercial solution, e.g., as advertised from Anaero Technology (www.anaero.co.uk/).

The application of the LAMACs may be of particular interest to researchers from various disciplines, not limited to bioprocess engineering and microbial ecology. Screening microbial communities for desired ecosystems functions tasks [18,37,38] or linking microbial processes to microbial community structure [20]. The system allows tackling common pitfalls with respect to the statistical evaluation [39] or experimental design [1], and at the same time provides a high-resolution automated sensing approach to monitor ecosystems functioning [40]. In this framework, LAMACs brings us one step further toward the understanding of the dynamic and function of complex microbial communities [41].

## Supporting information

S1 Fig Calibration of one peristaltic pump. The calibration was made with measuring different volumes of water after having rotated the pump one minute at different velocities.
(PDF)

S2 Fig Calibration of one pressure sensor. The calibration was made with a pressure imposed manually, read by the software and checked with a manometer.
(PDF)

S3 Fig Volatile fatty acid concentrations over time in the twelve reactors. Lines in light gray stand for INOC A, dark-gray stand for INOC B, and black stand for INOC C.
(PDF)

S4 Fig Picture of 5 LAMACs module. (PDF)

S5 Fig Bacterial abundances in 30 continuous reactors over a period of twelve weeks. (PDF)

S1 Table Equipment and detail cost for one LAMACs module. Supplier references are added from Table 1.
(PDF)

S2 Table Composition of the complex and simple synthetic media used. Complexity of the medium must be understood as the presence of polymers instead of monomers for the simple medium, and a greater number of monomer types as compared to the simple medium. The substrate composition of intermediate medium was added for the experiment detailed in S5 Fig.
(PDF)

S3 Table Comparison of biogas production rates. Four replicated reactors were used. Mean biogas production rates are expressed over the organic loading rate (ml∙gCOD^-1^). Kruskal Wallis tests (Chi^2^) were performed. When the test was significant (p-value < 0.05), the Dunn post-hoc test was applied to account for multiple comparisons of independent samples.
(PDF)
