# Supplementary material for: Multiplexed chemostat system for quantification of biodiversity and ecosystem functioning in anaerobic digestion
Source: PLoS One. 2018 Mar 8;13(3):e0193748. doi: 10.1371/journal.pone.0193748 (PMC5843216; doi:10.1371/journal.pone.0193748)
Supplement: S1 Fig — The calibration was made with measuring different volumes of water after having rotated the pump one minute at different velocities. (PDF) [file pone.0193748.s001.pdf]

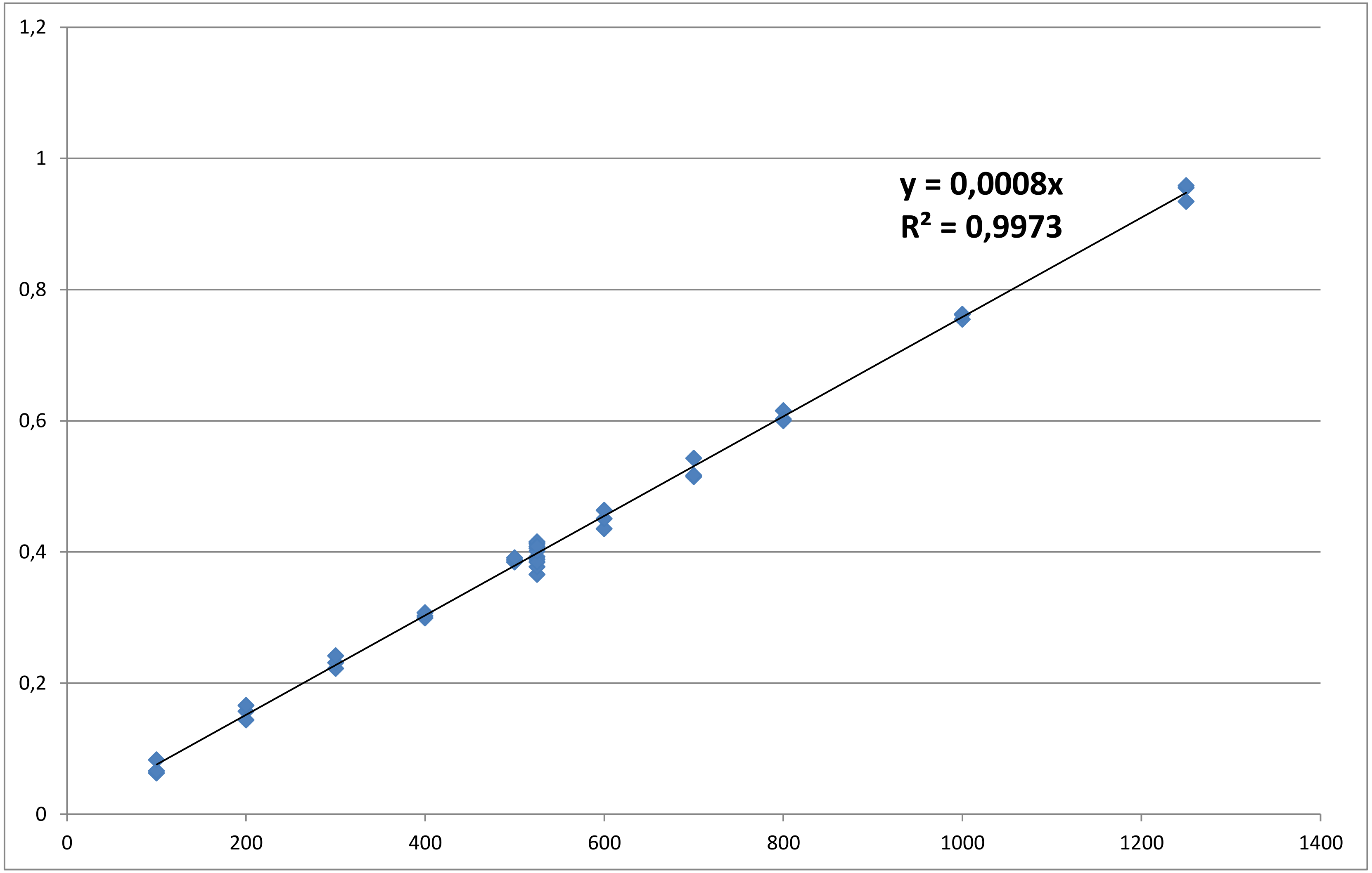

S1 Fig. Calibration of one peristaltic pump. The calibration was made with measuring different volumes of water after having rotated the pump one minute at different velocities.
