# Supplementary material for: Multiplexed chemostat system for quantification of biodiversity and ecosystem functioning in anaerobic digestion
Source: PLoS One. 2018 Mar 8;13(3):e0193748. doi: 10.1371/journal.pone.0193748 (PMC5843216; doi:10.1371/journal.pone.0193748)
Supplement: S2 Fig — The calibration was made with a pressure imposed manually, read by the software and checked with a manometer. (PDF) [file pone.0193748.s002.pdf]

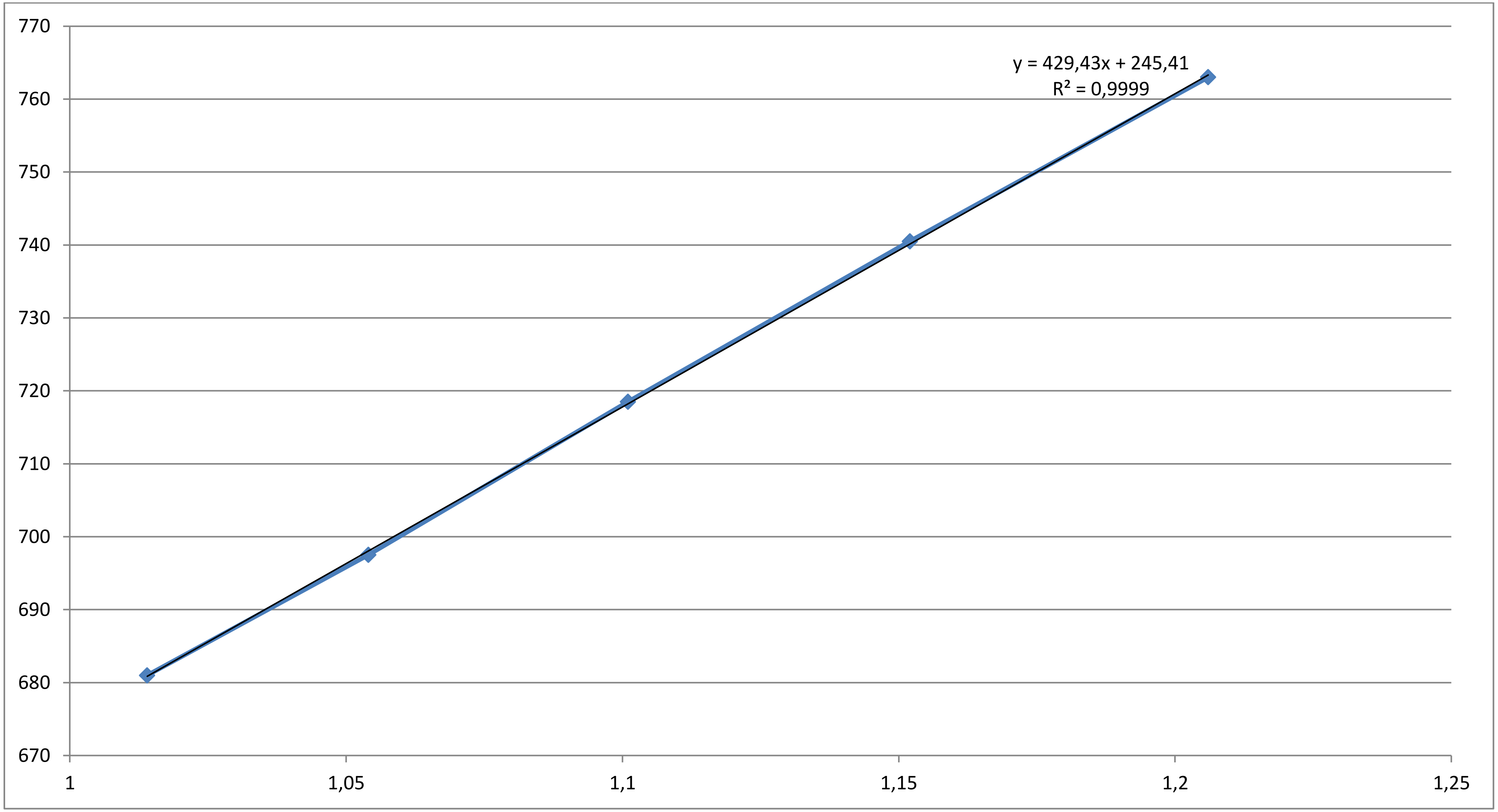

S2 Fig. Calibration of one pressure sensor. The calibration was made with a pressure imposed manually, read by the software and checked with a manometer.
