# Supplementary material for: Multiplexed chemostat system for quantification of biodiversity and ecosystem functioning in anaerobic digestion
Source: PLoS One. 2018 Mar 8;13(3):e0193748. doi: 10.1371/journal.pone.0193748 (PMC5843216; doi:10.1371/journal.pone.0193748)
Supplement: S3 Fig — Lines in light gray stand for INOC A, dark-gray stand for INOC B, and black stand for INOC C. (PDF) [file pone.0193748.s003.pdf]

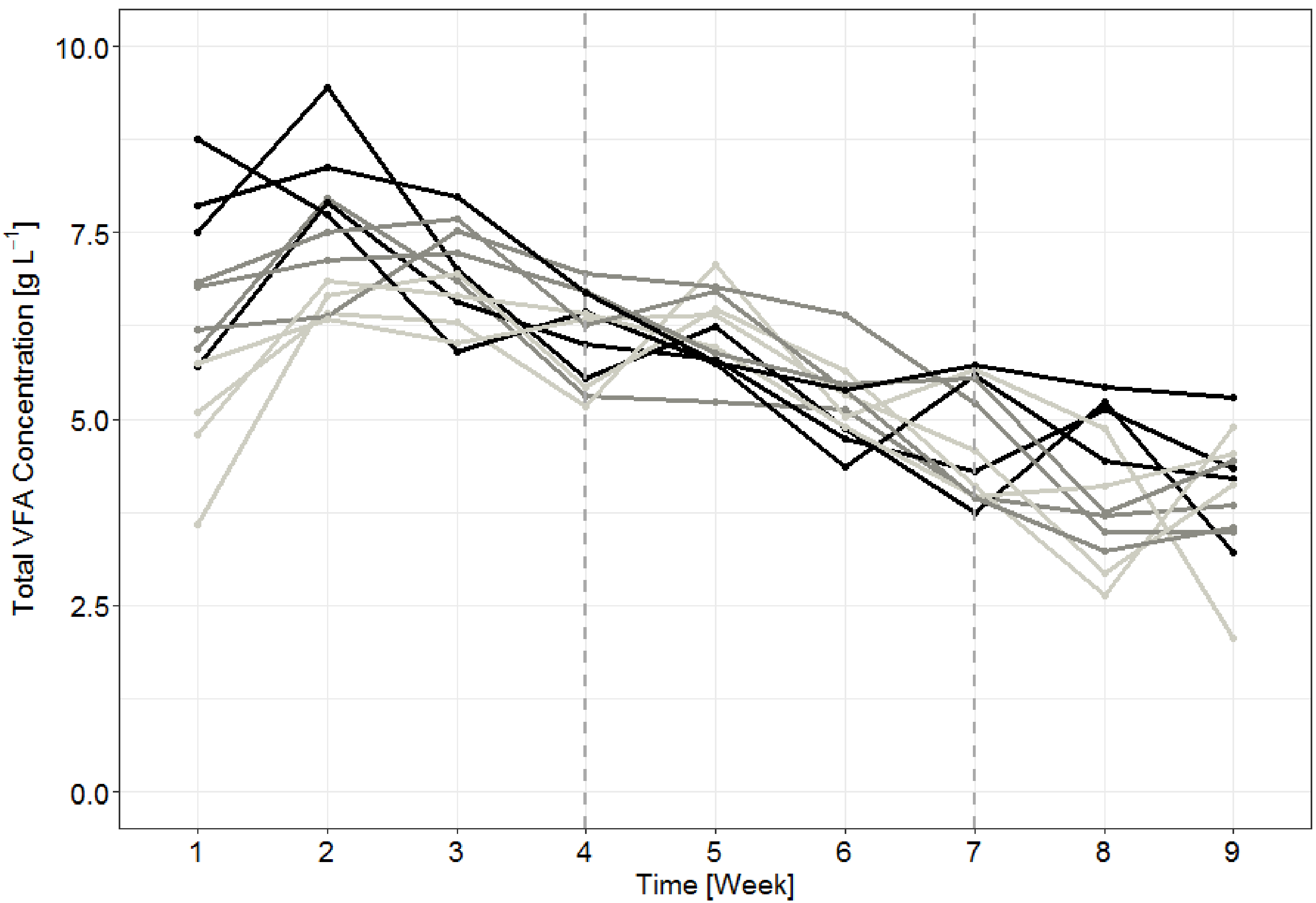

S3 Fig. Volatile Fatty Acid concentrations over time in the twelve reactors. Lines in light gray stand for INOC A, dark-gray for INOC B and black stand for INOC C
