# Supplementary figures and images for: Multiplexed chemostat system for quantification of biodiversity and ecosystem functioning in anaerobic digestion
Source: PLoS One. 2018 Mar 8;13(3):e0193748. doi: 10.1371/journal.pone.0193748 (PMC5843216; doi:10.1371/journal.pone.0193748)

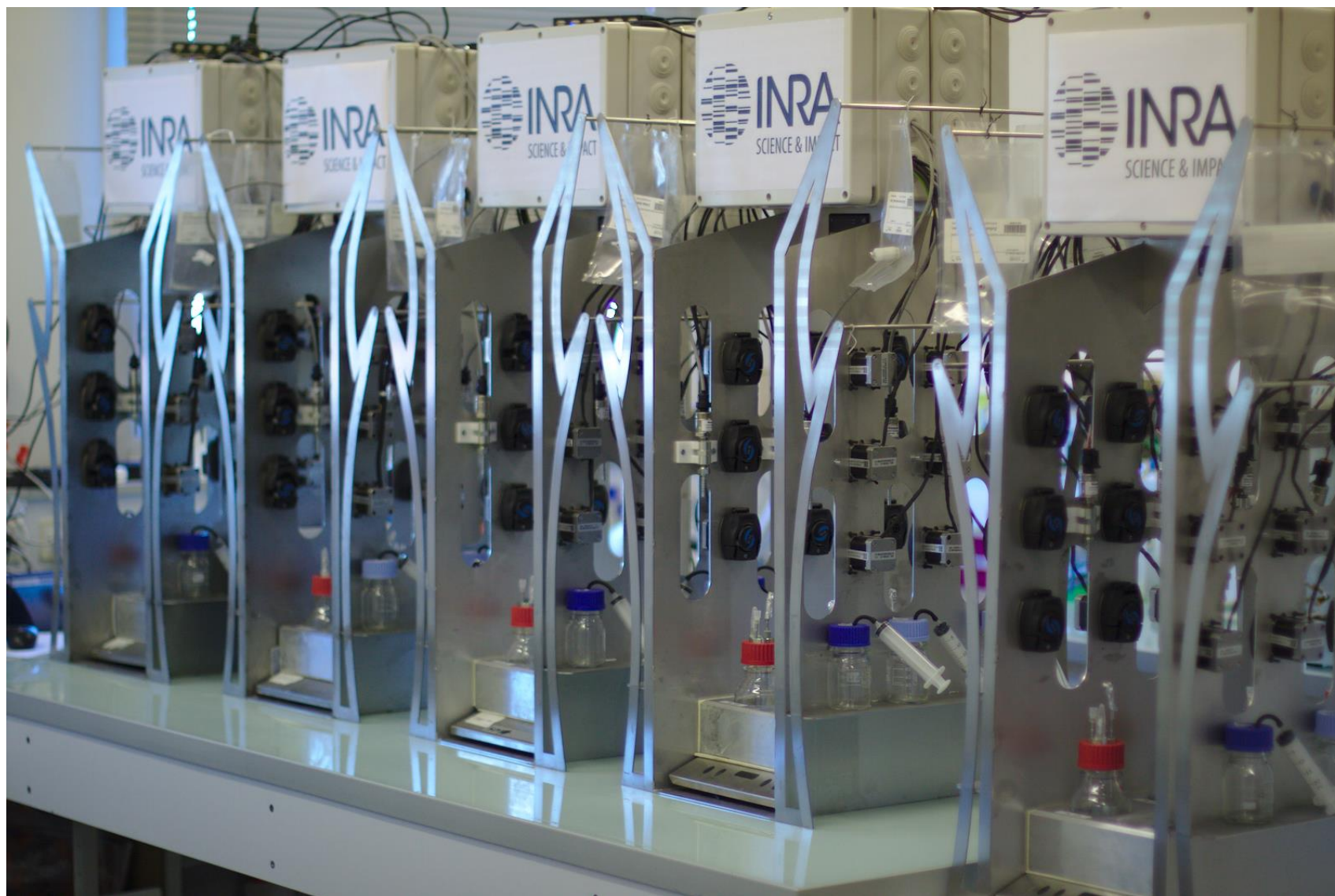

S4 Fig. Picture of 5 LAMACs module

Supplement: S4 Fig — (PDF) [file pone.0193748.s004.pdf]
