# Supplementary material for: Multiplexed chemostat system for quantification of biodiversity and ecosystem functioning in anaerobic digestion
Source: PLoS One. 2018 Mar 8;13(3):e0193748. doi: 10.1371/journal.pone.0193748 (PMC5843216; doi:10.1371/journal.pone.0193748)
Supplement: S5 Fig — (PDF) [file pone.0193748.s005.pdf]

Materials and methods

In another experiment, the system LAMACs was used to run 30 anaerobic digesters in parallel, therefore 5 modules. Four digestate from five different origins were sampled from pilot-scale and one from industrial scale. Digestates from these reactors were anaerobically conditioned for one month to allow the degradation of the remaining organic matter. They then were used to inoculate four replicated anaerobic bioreactors per inoculum at a concentration as volatile solids (VS) of 2 gVS·L^{-1}. All reactors were fed with liquid synthetic substrate of 10gCOD·L^{-1} concentration for twelve weeks. Three different substrates complexity as described in the supplementary table were used to feed ten reactors each. The reactors were in continuous mode, at a constant hydraulic retention time of 15 days over nine weeks under mesophilic conditions (37 °C). Biomass sampling was done every week. One day of biomass wasting (12mL) were collected and centrifuged 10 min at a G-force set between 3.550 and 7.140g depending on the biomass concentration. After removing 9mL of the supernatant, pellet was resuspended and aliquots of 500µL were sampled in 2mL sterile Eppendorf tubes and stored at -20°C. For DNA extraction, FastDNA SPIN kits for soil (MP Biomedicals, Santa Ana, USA) were used according to the manufacturer's instructions. Purified DNA was conserved at -20°C in 100 µL of molecular grade purity water until further use. For quantitative PCR Bacteria 16rRNA amplification, the primers W208 and W209 [1] were used: W208 F338-354 5'-ACTCC TACGG GAGGC AG-3' at 100nMf; and W209 R805-536 5'-GACTA CCAGG GTATC TAATC C-3' at 250nMf. The probe was Taqman Tamra W210 F516-536 5'-Yakima Yellow-TGCCA GCAGC CGCGG TAATA C-Tamra-3' at 50nMf. For the amplification of bacterial sequences, the PCR mixture contained 6.5 µL Mix Biorad SsoAdvanced Universal Probes Supermix (Bio-rad, Hercules, United States), 0.5 µL of each primers and probe, 2.5 µL water and 2 µL of DNA extracts for a total volume of 12.5 µL. All samples were run at two dilutions in duplicate on a CFX96 (Bio-Rad Hercules, United States) qPCR machine using a program with 2 minutes at 95°C enzyme activation followed by 40 cycles of 7 s at 95°C for dissociation and 25 s at 60°C for hybridization and elongation.

Bacteria abundances of the 30 reactors are displayed in the figure below. Orange lines stand for *Bacteria* abundances of reactors fed with complex substrate, green for simple substrate and blue for intermediate substrate.

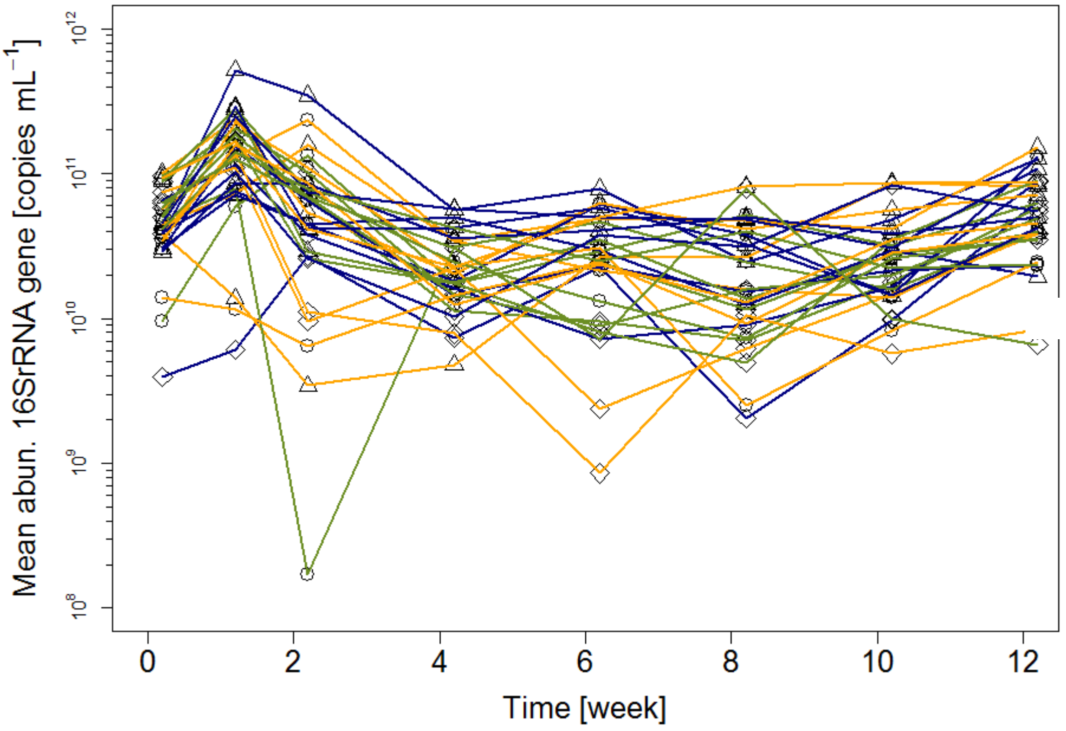

S5 Fig. Bacterial abundances in 30 continuous reactors over a period of twelve weeks.
