# Supplementary material for: Multiplexed chemostat system for quantification of biodiversity and ecosystem functioning in anaerobic digestion
Source: PLoS One. 2018 Mar 8;13(3):e0193748. doi: 10.1371/journal.pone.0193748 (PMC5843216; doi:10.1371/journal.pone.0193748)
Supplement: S1 Table — Supplier references are added from Table 1. (PDF) [file pone.0193748.s006.pdf]

	Equipment for one module LAMACs -6reactors	Supplier reference	Supplier	cost for 6 reactors (€)
**bottling**	6 Stainless steel three-hose connectors	custom made	Garaud, Carcassonne, France	**222**
	250 mL bottles, cap connectors, magnetic stirrer	laboratory equipment	-	39
			subtotal	**261**
**temperature regulation**	Heating bloc	custom made	Garaud, Carcassonne, France	807
	Temperature regulator	custom made	YESSS Electrique, Francheville, France	140
			subtotal	**947**
**feeding and biomass wasting**	12 peristaltic pump with stepping motors (FZ10)	PPELEA02204	A2V Flowtronique, Gazeran, France	1320
	Controller module (TMCM 6110)	VARTRI00914	A2V Flowtronique, Gazeran, France	615
	Motors power supply	-	YESSS Electrique, Francheville, France	80
			subtotal	**2015**
**pressure measurement**	6 peristaltic pump with stepping motors (FZ10)	PPELEA02204	A2V Flowtronique, Gazeran, France	660
	6 Pressure sensor (PX2EN1XX050PAAAX, Honeywell)	853-6471	RS components Corby, UK	380
	Pressure sensor power supply	-	YESSS Electrique, Francheville, France	50
			subtotal	**1090**
**unit assembly**	Chassis	custom made	Garaud, Carcassonne, France	707
	Electric jacket	-	YESSS Electrique, Francheville, France	30
	Laptop	-	-	600
			subtotal	**1337**
**mixing**	Magnetic stirring plate (Variomag Multipoint 6)	3302060	Sodipro, Echirolles, France	**1060**
			**Total**	**6710**

S1 Table. Equipment and detail cost for one LAMACs module. Supplier references are added from Table 1.
