# Supplementary material for: Multiplexed chemostat system for quantification of biodiversity and ecosystem functioning in anaerobic digestion
Source: PLoS One. 2018 Mar 8;13(3):e0193748. doi: 10.1371/journal.pone.0193748 (PMC5843216; doi:10.1371/journal.pone.0193748)
Supplement: S2 Table — Complexity of the medium must be understood as the presence of polymers instead of monomers for the simple medium, and a greater number of monomer types as compared to the simple medium. The substrate composition of intermediate medium was added for the experiment detailed in S5 Fig. (PDF) [file pone.0193748.s007.pdf]

Substrate		Formula	[monomer]	Concentration [$g{\cdot}l^{-1}$]	CAS Number	Reference used (if not Sigma)
Complex medium	Yeast nitrogen base		-	1.07		Y0626
	Polyethylene glycol (MW 20.000)	$H(C_2H_4O)nOH$	[Ethylen glycol]$_n$	1.176	25322-68-3	P2139
	Carboxymethyl cellulose	$[C_{28}H_{30}Na_8O_{27}]$ n	[Glucose]$_n$	4.04	9004-32-4	C5678
	Starch	$[C_6H_{10}O_5]$n	[Glucose]$_n$	2.963	9005-25-8	S2004
	Sucrose	$[C_{12}H_{22}O_{11}]$n	[Glucose-Fructose]	2.083	57-50-1	84100
	Inuline	$C_6nH_{10n}+2O_{5n+1}$	[Fructose]$_n$	1.408	9005-80-5	myprotein.com
	Malic acid	$C_4H_6O_5$		1.235	6915-15-7	myprotein.com
	Lactose 1·H_20	$C_{12}H_{22}O_{11}$. · H_20	[Glucose-Galactose]	2.198	5989-81-1	L3625
	Trehalose 2·H_20	$C_{12}H_{22}O_{11}$ · 2H_20	[Glucose]$_n$	0.667	6138-23-4	T9531
	Raffinose 5· H_20	$C_{18}H_{32}O_{16}$ · 5H_2O	[Glucose-Galactose-[Fructose]	0.33	17629-30-0	R0250
	Itaconic acid	$C_5H_6O_4$		0.297	97-65-4	I29204
	Glycerol phosphate disodium salt · H_20	$C_3H_7Na_2O_6P$ · xH_2O		5.455	55073-41-1	G6501
	α-D glucopyranoside	$C_7H_{14}O_6$		0.583	97-30-3	66940
	Diethyl malate	$C_8H_{14}O_5$		0.397	03/12/7554	W237418
Simple medium	Yeast nitrogen base			0.542	Y0626	Y0626
	Ethylen glycol	$C_2H_6O_2$	Ethylen Glycol	1.524	107-21-1	324558
	Glucose	$C_6H_{12}O_6$	Glucose	4.261	50-99-7	G8270
	Fructose	$C_6H_{12}O_6$	Fructose	4.213	57-48-7	F0127
Intermediate substrate for suppl. figure	Yeast nitrogen base	-		0,54		Y0626
	Polyethylene glycol (MW 8.000)	H(C2H4O)n OH	[Ethylen glycol]n	1,47	2065419	W237418
	Dextrin	C6H12O6	[Glucose]n	2,58	9004-53-9	31405
	Inuline	C6nH10n+2O5n+1	[Fructose]n	1,76	9005-80-5	myprotein.com

S2 Table. Composition of the complex and simple synthetic media used. Complexity of the medium must be understood as the presence of polymers instead of monomers for the simple medium, and a greater number of monomer types as compared to the simple medium. The substrate composition of intermediate medium was added for the experiment detailed in **S5** Fig.
