# Supplementary material for: Multiplexed chemostat system for quantification of biodiversity and ecosystem functioning in anaerobic digestion
Source: PLoS One. 2018 Mar 8;13(3):e0193748. doi: 10.1371/journal.pone.0193748 (PMC5843216; doi:10.1371/journal.pone.0193748)
Supplement: S3 Table — Four replicated reactors were used. Mean biogas production rates are expressed over the organic loading rate (ml∙gCOD-1). Kruskal Wallis tests (Chi2) were performed. When the test was significant (p-value < 0.05), the Dunn post-hoc test was applied to account for multiple comparisons of independent samples. (PDF) [file pone.0193748.s008.pdf]

Week	INOC A	INOC B	INOC C	Chi-2	p-value	pairwise comparison
1	110.1 ± 39.5	177.9 ± 107.5	249.2 ± 163.0	32.96	6.9e-08	INOC A different from INOC B and C
2	265.9 ± 144.9	331.0 ± 177.3	327.0 ± 107.8	18.49	9.7e-05	INOC A different from INOC B and C
3	217.9 ± 106.8	85.8 ± 42.8	270.9 ± 34.4	199.57	2.2e-16	All pairwise different
4	141.2 ± 109.1	48.3 ± 47.0	116.9 ± 42.5	108.04	2.2e-16	INOC B different from INOC A and C
5	40.8 ± 16.8	29.9 ± 7.5	114.8 ± 34.9	237.43	2.2e-16	All pairwise different
6	82.8 ± 39.5	37.7 ± 10.8	92.4 ± 22.3	179.52	2.2e-16	All pairwise different
7	163.3 ± 72.5	113.7 ± 61.7	174.5 ± 66.5	48.87	2.5e-11	All pairwise different
8	175.2 ± 78.3	200.7 ± 75.9	233.3 ± 57.9	37.75	6.3e-09	INOC C different from INOC A and B
9	243.9 ± 69	268.2 ± 123.4	257.9 ± 38.6	3.34	0.1886	-

S3 Table. Comparison of biogas production rates. Four replicated reactors were used. Mean biogas production rates are expressed over the organic loading rate (ml·gCOD-1). Kruskal Wallis tests (Chi2) were performed. When the test was significant (p-value < 0.05), the Dunn post-hoc test was applied to account for multiple comparisons of independent samples.
